# Observation of Influence of Cataract Surgery on the Ocular Surface

**DOI:** 10.1371/journal.pone.0152460

**Published:** 2016-10-03

**Authors:** Yuli Park, Hyung Bin Hwang, Hyun Seung Kim

**Affiliations:** 1 Department of Ophthalmology, Yeouido St. Mary’s Hospital, College of Medicine, The Catholic University of Korea, Seoul, Korea; 2 Department of Ophthalmology, Incheon St. Mary’s Hospital, Seoul, Korea; Medical University Graz, AUSTRIA

## Abstract

**Introduction:**

To evaluate meibomian gland function, changes of lacrimal tears and ocular surface parameters and tear inflammatory mediators following cataract surgery.

**Methods:**

48 eyes of 34 patients who underwent uncomplicated phacoemulsification were involved and divided into 2 groups with those who had preexisting dry-eye before cataract surgery and those who did not. Ocular symptom score, Schirmer I test, tear film break-up time (TBUT), corneal sensitivity threshold, corneal staining, inflammatory cytokine activities, lid margin abnormalities, meibum expressibility, meibum quality and meibomian gland imaging were evaluated preoperatively, at 1 day, 1 and 2 months postoperatively.

**Results:**

Ocular symptom scores were worse at 1 and 2 months postoperatively but, TBUT, corneal staining score and corneal sensitivity threshold showed gradual improvements at 1 month and 2 months postoperatively (*p*<0.05, respectively). Interestingly there were statistically significant improvements in TBUT, corneal staining score and corneal sensitivity threshold at 1 month postoperatively when topical eye drops were used compared to the period without topical therapy which is the months 2 postoperatively. There were statistically significant decreases in IL-1β, IL-6, IL-8, MCP-1, TNF-α and IFN-γ concentrations at 1 and 2 months postoperatively. Lid margin abnormalities, meibum quality and expressibility scores increased significantly (*p* < 0.05, respectively) at postoperative period. Compared with the no dry eye group, dry eye group revealed significantly higher ocular symptom scores, lower TBUT, higher lid margin abnormalities, meibum quality and expressibility scores after cataract surgery. There were significant correlations between IL-6 and parameters of dry eye, and between MGD parameters and ocular symptom scores.

**Conclusions:**

Our study revealed that meibomian gland function is influenced after cataract surgery accompanying structural changes and these were correlated with increased ocular symptom scores. Therefore, it could elucidate the development of dry eye related to cataract surgery.

## Introduction

Many patients have complained of dry eye and symptoms of irritations after cataract surgery and it has been shown that both incidence and severity of dry eye increase [1]. In particular, the reduction in tear break up time (TBUT) and squamous metaplasia in conjunctival impression cytology were documented after phacoemulsification [2]. The exacerbation of dry eye after cataract surgery is possibly multifactorial. The reduced corneal sensitivity due to the transection of the corneal nerves [3], phototoxic damage from an exposure to the microscopic light [4], multiple irrigations of the corneal epithelium during operation [5], elevation of inflammatory cytokines in the lacrimal tear [6], and the use of topical anesthesia and antibiotics during surgery in addition to preservative containing topical eye drops administered after surgery [7] would influence dry eye following cataract surgery, but how these factors influence dry eye after cataract surgery is unclear. Kim et al. reported benzalkonium chloride induces tear film instability and ocular surface damage in a dose dependent manner [8]. Povidone iodine used before surgery for sterilization could possibly induce tear film instability and ocular surface damage in a time dependent manner which is under our current study. Moon et al. showed that an aspirating speculum could aggravate dry eye after cataract surgery [9]. Increased levels of inflammatory cytokines were detected in the tears of dry eye patients and the immune-pathological changes were shown in the conjunctiva of dry eye patients [10]. Few studies have investigated changes in the levels of the inflammatory cytokines before and after cataract surgery and few reports have focused on the development of meibomian gland dysfunction (MGD) following cataract surgery [11]. Still, we could face some patients who complain of ocular discomfort, yet lack objective signs, such as ocular surface staining after cataract surgery.

The aim of this study is to investigate whether cataract surgery affects meibomian gland dysfunction, and to evaluate associated changes in dry eye parameters including lacrimal tear inflammatory cytokines following cataract surgery.

## Materials and Methods

This study was conducted in accordance with the institutional review board regulations (IRB), investigator obligations, and adhered to the tenets of the Declaration of Helsinki. All patients were fully informed of the details and possible risks of the procedure, and written informed consents were obtained from all the participated patients. This study was reviewed and approved by the Institutional Review Boards (IRBs) of Yeouido St. Mary’s Hospital, College of Medicine, the Catholic University of Korea (IRB#SC14RISI0160).

### Study population and procedures

48 eyes of 34 patients meeting the inclusion and exclusion criteria were evaluated before and after cataract surgery, between May 2014 and Aug. 2014 in our clinic. Subjects were divided into two groups based on the signs and symptoms of dry eye using the DEWS criteria before cataract surgery [12,13]. Criteria for diagnosis of dry eye included symptom severity score > 20 and tear film break-up time (TBUT) < 7 seconds[12,13]. Patients were excluded if they were using contact lens, ocular surgery in the past year, the history of ocular injury, infection, or other ocular surface diseases. Exclusion criteria also included continuous use of topical ocular medications such as steroids, antibiotics, drugs with preservation, and systemic drugs that would influences tear film.

### Cataract surgery

One surgeon performed all cataract surgeries. 2.85mm sized clear corneal incision was made and the phacoemulsification time was 1 to 2 minutes. An intraocular lens was inserted in the posterior chamber. In all cases, no intraoperative complication happened. Patients instilled 0.5% moxifloxacin 0.5% (Vigamox^Ⓡ^, Alcon, Fort Worth, TX, USA) and 1% prednisolone acetate (Pred Forte^®^, Allergan, Irvine, CA, USA) eye drops four times daily for 1 month postoperatively.

### Ocular symptom score

All patients completed ocular surface disease index (OSDI) questionnaire and had meibomian gland and lacrimal tear evaluations performed by the same investigator. We eliminated item 4 and 5, which evaluated the presence of blurred vision, because it was hard to discriminate the change of symptoms caused by cataract surgery alone or combined with visual symptoms due to dry eye that was induced by phacoemulsification. Subjective symptoms were graded on a numerical scale from 0 to 4 according to the OSDI score [14]. We rated the intensity of dry eye symptoms from 0 to 4 as follows: 0, none; 1, mild; 2, moderate; 3, severe; 4, very severe. The frequency of dry eye symptoms was quantified as follows: 0, none; 1, some of the time; 2, half of the time; 3, most of the time; 4, all the time. Aggravation of dry eye was quantified as follows: 0, none; 1, mild; 2, moderate; 3, severe; 4, very severe. The total score of dry eye symptoms was calculated as follows: (intensity score + frequency score+ aggravation score) divided by 3. Scores ranged from 0 to 4, with higher scores demonstrating severe symptoms [15].

### Clinical evaluation of lacrimal tear & ocular surface

The Bio-microscopic examinations consisted of TBUT, corneal and conjunctival staining.

To measure TBUT, 5μl of 2% fluorescein solution was instilled in the conjunctival sac, and the integrity of the tear film was monitored and measured up to the time until one or more dry spots appeared in the precorneal tear film from the last blink by slit-lamp microscopy.

Schirmer I test was performed by bending the Schirmer strip at the notch, and placing the strip beneath the temporal lid margin. After 5 minutes, the strip was removed and measured to the point of maximum wetting.

Corneal fluorescein staining was examined through slit-lamp evaluation with cobalt blue illumination. The staining was measured for each of the five regions of the cornea: central, superior, temporal, nasal, and inferior. The degree of the staining was based on the following: grade 0, no staining; grade 1, superficial stippling and micro-punctate staining; grade 2, macro-punctate staining with some coalescent areas; and grade 3, numerous coalescent macro-punctate areas. Each of the five regions was graded on a scale from 0 to 3. The scores of the five areas were added to obtain a total score for each eye [16].

Corneal sensitivity was measured with a Cochet–Bonnet esthesiometer (Luneau Ophthalmologie, Chartres, France). The cornea was touched smoothly by the nylon filament.

All measurements were performed by the same investigator at preoperatively, 1 day, 1 and 2 months postoperatively.

### Lacrimal tear sample and multiplex immunobead analysis

The unstimulated lacrimal tear was accumulated from the inferior tear meniscus by 0.5 μl glass capillary micropipette (Drummond, Broomall, Pennsylvania, USA). The lacrimal tears were centrifuged for two minutes and immediately transported to a -80°C freezer. Cytokines and chemokines were analyzed using a commercial assay system of immunoassay kits and panels using a magnetic bead-based immunoassay kit (Luminex 200; Luminex Corp., Austin, TX). The quantified cytokines and chemokines were IL-8, IL-6, IL-1β, TNF-α, MCP-1 and IFN-γ. To calculate molecular concentrations of tear cytokines, we analyzed the median fluorescent intensity data using a 5-parameter logistic or spline curve-fitting method.

### Clinical evaluation of meibomian gland

The diagnosis of the MGD was made by the dysfunction (lack of expressible meibum from > 75% of glands) and the appearance of two or more morphologic changes of the meibomian glands, including irregularity of the lid margin, orifice metaplasia, and acinar atrophy. Lid margin abnormalities including plugged meibomian gland orifices, vascular engorgement, anterior or posterior displacement of the mucocutaneous junction, and irregular lid margin were scored from 0 through 4 according to the number of these abnormalities present in each eye [17].

The expressibility of the meibum was scored by the application of the digital pressure to the upper tarsus, and the degree of ease with which the meibum was induced was evaluated semiquantitatively as follows: 0, clear meibum easily expressed; 1, cloudy meibum expressed with mild pressure; 2, cloudy meibum expressed with more than moderate pressure; and 3, meibum not expressed, even with the hard pressure [18].

The quality of the meibum was scored by the digital pressure over 8 meibomian glands of the lower lids. The meibum secretion was graded as follows: 0, clear; 1, cloudy; 2, cloudy with granular debris; and 3, thick like toothpaste.

Using the noncontact meibography system that consists of an infrared transmitting filter (IR-83, Hoya, Tokyo, Japan) and an infrared charge coupled video camera (XC-EI50, Sony, Tokyo, Japan), the meibomian glands were observed. Partial or complete loss of the meibomian glands was scored using the following grades for each eyelid: 0, no loss of the meibomian glands; 1, lost area was less than one third of the total area of the meibomian glands; 2, lost area was between one third and two thirds of the total area of the meibomian glands; and 3, lost area was over two thirds of the total area of the meibomian glands. Meibo-scores for the upper and lower eyelids were summed to obtain a score from 0 through 6 for the each eye [19].

### Statistical analysis

SPSS software (version 13.0, SPSS Inc., Chicago, IL) was used for statistical analysis. A linear mixed model with Bonferroni post hoc analysis was used to evaluate repeated measurements of continuous values. Pairwise comparisons of group categorical variables were done using the Mann-Whitney U test. Wilcoxon signed rank test was used to compare within-group categorical variable changes from the baseline. The repeated measures analysis of variance was used to compare within-group continuous variable changes from the baseline. *P* < 0.05 was considered statistically significant.

## Results

There was no statistically significant difference between the two groups before cataract surgery, except TBUT, Schirmer I test and corneal staining score. Of the total 34 patients, dry eye group included 18 patients, and no dry eye group included 16 patients. In the dry eye group, 12 were female, and the mean age ± standard deviation (SD) was 67.24 ± 5.91 years. In the no dry eye group, 9 were female and the mean age was 64.18 ± 6.02 years. There were no significant differences of sex and age between the two groups.

### Ocular symptom score

**Fig 1:**
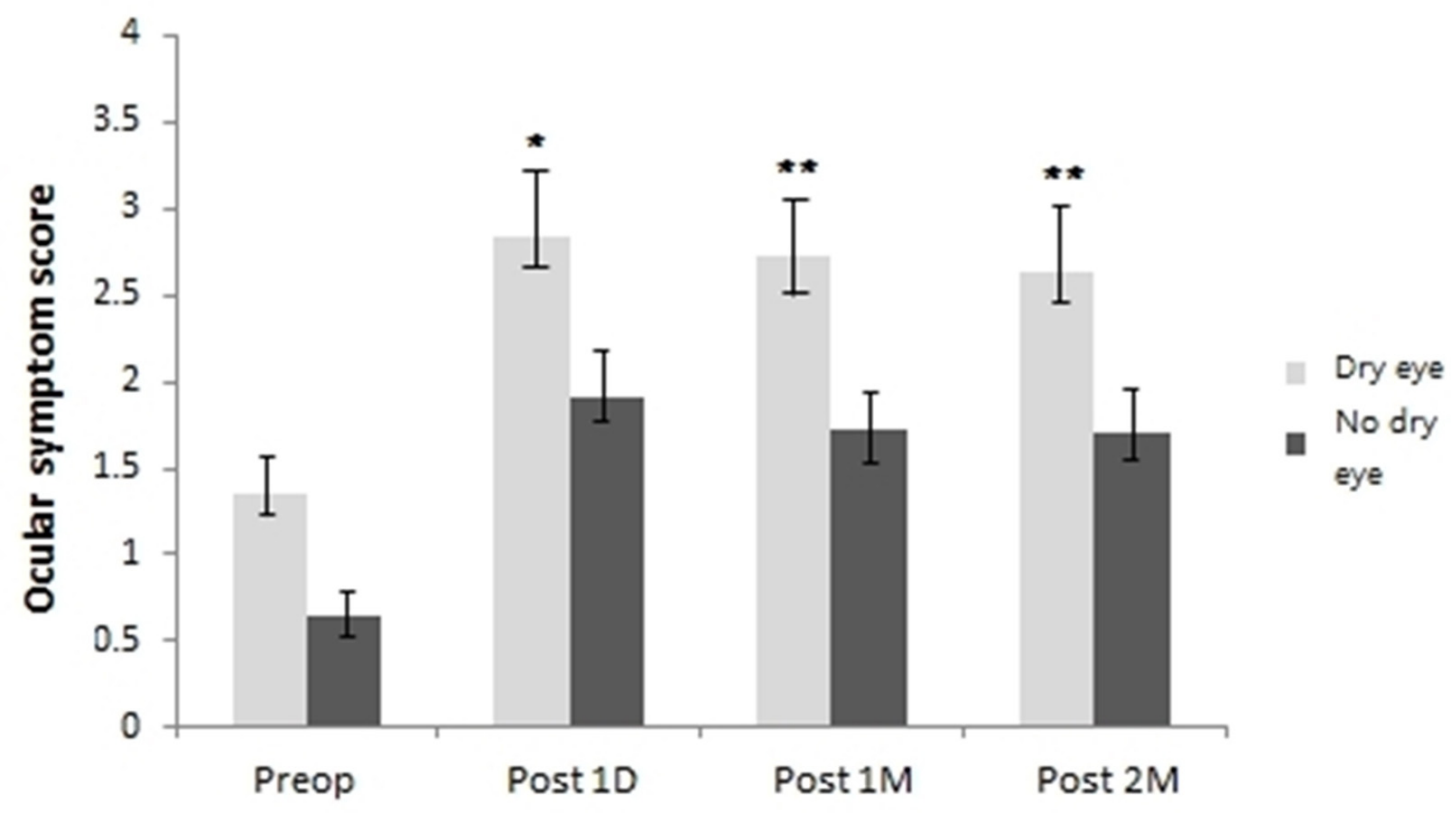
Change in ocular symptom score from preoperative value. The increase in ocular symptom score was statistically significant in the dry eye group at 1 day, 1 and 2 months postoperatively compared to the no dry eye group (*P* <0.05). Each value represents the mean ± standard error of the mean (SEM). Significance was evaluated with random-effects analysis of variance (ANOVA) model; p values of <0.05 were considered statistically significant. ***P < .001, **P < .01, *P < .05, using a linear mixed model with post hoc analysis, or generalized estimating equations model.

The symptom scores were significantly worse in the dry group than in the no dry eye group at 1 day (2.84 ± 0.81, 1.91 ± 0.70, respectively), 1 month (2.72 ± 0.69, 1.73 ± 0.64, respectively) and 2 months postoperatively (2.64 ± 0.71, 1.71 ± 0.61, respectively) (*P* <0.05, respectively) (Fig 1). The decrease in symptom score was not significant at 1 and 2 months after cataract surgery in dry eye group but, it was statistically significant in no dry eye group at 2 months after cataract surgery (*P*<0.05).

### Evaluation of lacrimal tear & ocular surface

#### 1) TBUT and Schirmer I test

**Fig 2:**
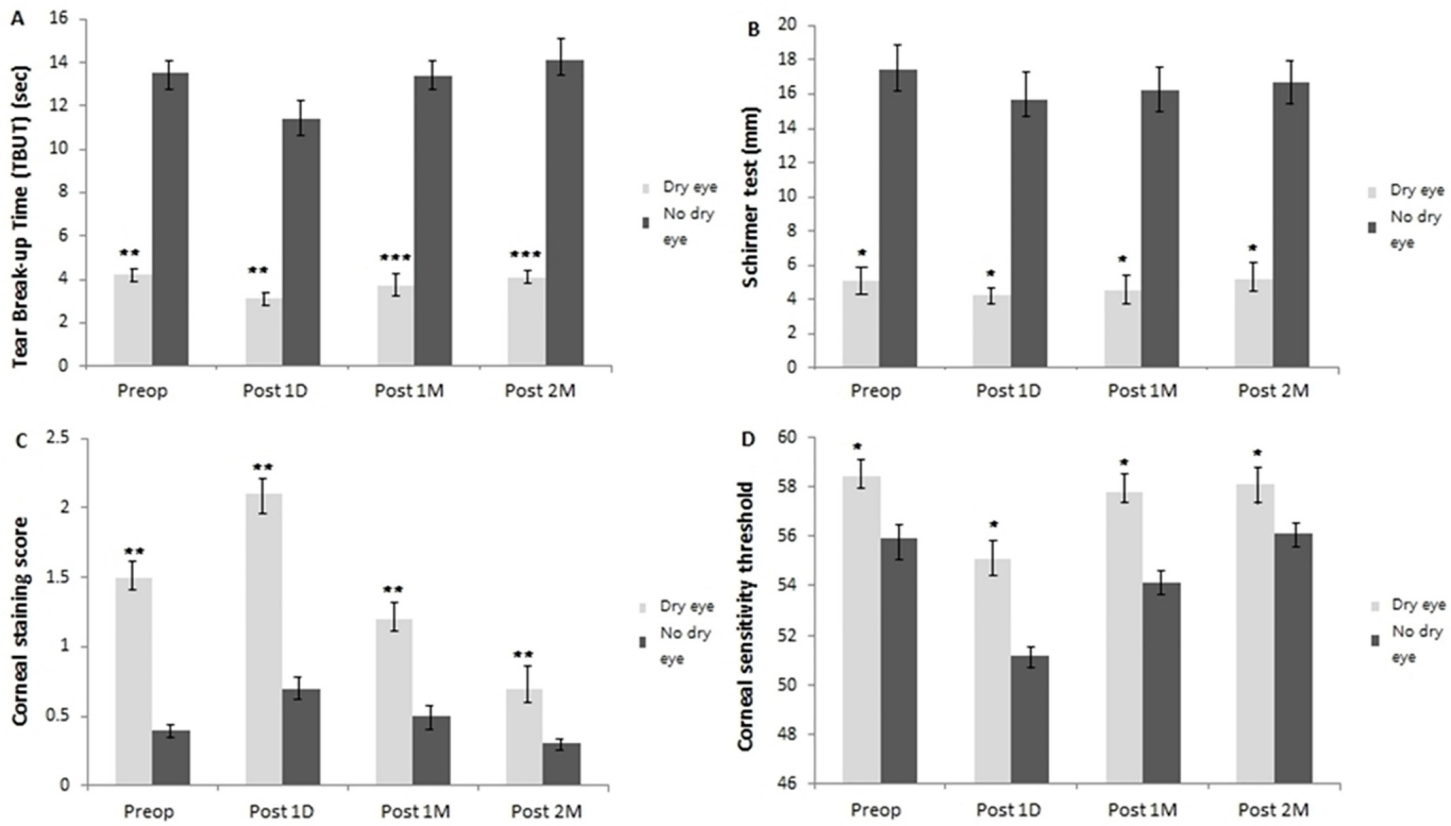
Changes in lacrimal tear and ocular surface parameters preoperatively, 1 day, 1 and 2 months postoperatively from preoperative value. (A) Change in TBUT (sec) from preoperative value. TBUT was significantly short in the dry eye group at 1 day, 1 and 2 months postoperatively compared to the no dry eye group (*P* <0.05). (B) Change in Schirmer I test (mm) from preoperative value. Schirmer I test value was significantly short in the dry eye group at 1 day, 1 and 2 months postoperatively compared to the no dry eye group (*P* >0.05). (C) Change in corneal staining score from preoperative value. There was a statistically significant decrease in the corneal staining score at 1 and 2 months postoperatively but the dry eye group showed higher score than the no dry eye group (*P* <0.05). (D) Change in corneal sensitivity threshold (mm) from preoperative value. Corneal sensitivity threshold slowly recovered to preoperative value at 1 and 2 months postoperatively but the dry eye group showed higher threshold level than the no dry eye group (*P* <0.05). Each value represents the mean ± standard error of the mean (SEM). Significance was evaluated with random-effects analysis of variance (ANOVA) model; p values of <0.05 were considered statistically significant. ***P < .001, **P < .01, *P < .05, using a linear mixed model with post hoc analysis, or generalized estimating equations model.

There was a statistically significant improvement in TBUT at 1 month (13.4 ± 2.7, 3.7 ± 0.5, respectively) and 2 months (14.1 ± 3.1, 4.1 ± 0.4, respectively) postoperatively in the no dry eye group compared to the dry eye group (*P* <0.001, respectively) (Table 1, Fig 2A). TBUT was more significantly worsened in the dry eye group compared to the no dry eye group at 1 day postoperatively and the recovery was significantly slower in the dry eye group than no dry eye group at 1 and 2 months postoperatively. Interestingly there was a statistically significant improvement in recovery at 1 month postoperatively when topical eye drop was used compared to the period without topical therapy which is the months 2 postoperatively.

**Table 1:** Mean value of ocular surface parameters measured preoperatively, at 1 day, 1 and 2 months after cataract surgery.

**Parameters**	**Dry eye group**							
	**Preoperative value**	**1 day**	**1 month**	**2 months**	**Overall**	**Preop. Vs 1 day**	**Preop. vs 1 month**	**Preop. vs 2 months**
**TBUT (sec)**	4.2±0.4	3.1±0.3	3.7±0.5	4.1±0.4	*<0*.*001*	*<0*.*001*	*<0*.*001*	*<0*.*001*
**Schirmer test (mm)**	5.1±0.5	4.3±0.5	4.5±0.8	5.2±0.6	*0*.*894*	*0*.*816*	*0*.*924*	*0*.*557*
**Corneal sensitivity threshold (mm)**	58.4±1.7	55.1±1.8	57.8±1.5	58.1±1.7	*0*.*016*	*0*.*024*	*0*.*021*	*0*.*019*
**Corneal staining score**	1.5±0.4	2.1±0.8	1.2±0.7	0.7±0.3	*0*.*024*	*0*.*041*	*0*.*031*	*0*.*035*
	**No dry eye group**							
**TBUT (sec)**	13.5±2.5	11.4±2.3	13.4±2.7	14.1±3.1	*<0*.*001*	*<0*.*001*	*<0*.*001*	*<0*.*001*
**Schirmer test (mm)**	17.4±5.9	15.7±4.8	16.2±5.1	16.7±5.4	*0*.*829*	*0*.*529*	*0*.*241*	*0*.*268*
**Corneal sensitivity threshold (mm)**	55.9±1.4	51.2±0.9	54.1±1.2	56.1±1.3	*0*.*295*	*0*.*262*	*0*.*418*	*0*.*350*
**Corneal staining score**	0.4±0.1	0.7±0.2	0.5±0.1	0.3±0.1	*0*.*031*	*0*.*035*	*0*.*029*	*0*.*027*

TBUT = tear film break-up time

Continuous values were analyzed by linear mixed model with Bonferroni post hoc analysis.

Non-continuous values were analyzed by generalized linear mixed model analysis with Bonferroni post hoc analysis.

Schirmer I score was worsened in both groups at 1 day postoperatively although the values were not statistically significant (Fig 2B). It was significantly improved at 1 month (16.2 ± 5.1, 4.5 ± 0.8, respectively) and 2 months (16.7 ± 5.4, 5.2 ± 0.6, respectively) postoperatively in the no dry eye group compared to the dry eye group (Table 1). The difference of the recovery was not statistically significant in both groups at 1 and 2 months postoperatively (*P* >0.05, respectively). There was not a statistically significant improvement in recovery at 1 month postoperatively when topical eye drop was used compared to the period without topical therapy which is the months 2 postoperatively.

#### 2) Corneal staining score

There was a statistically significant decrease in the corneal staining score at 1 month (0.5 ± 0.1, 1.2 ± 0.7, respectively), 2 months (0.3 ± 0.1, and 0.7 ± 0.3, respectively) postoperatively in the no dry eye group compared to the dry eye group (*P* <0.05, respectively) (Table 1, Fig 2C). It was more significantly worsened in the dry eye group compared to the no dry eye group at 1 day postoperatively. Interestingly there was a statistically significant improvement in the recovery at 1 month postoperatively when topical eye drop was used, compared to the period without topical therapy which is the months 2 postoperatively in both groups.

#### 3) Corneal sensitivity threshold

Corneal sensitivity threshold was significantly lower at 1 day postoperatively (55.1 ± 1.8, 51.2 ± 0.9, respectively) than it was preoperatively (58.4 ± 1.7, 55.9 ± 1.4, respectively) in both dry and no dry eye groups (Table 1). Corneal sensitivity threshold was more slowly recovered in the dry group than in the no dry eye group at 1 month (57.8 ± 1.5, 54.1 ± 1.2, respectively) and 2 months postoperatively (58.1 ± 1.7, 56.1 ± 1.3, respectively) (*P* <0.05, respectively) (Fig 2D). It was worsened in both groups at 1 day postoperatively and the recovery was significantly slower in the dry eye group than no dry eye group at 1 and 2 months postoperatively. Interestingly there was a statistically significant improvement in recovery of corneal sensitivity threshold at 1 month postoperatively when topical eye drop was used compared to the period without topical therapy which is the months 2 postoperatively.

### Inflammatory cytokines in lacrimal tears

In the no dry eye group, the initial concentrations of IL-1β, IL-6, IL-8, MCP-1, TNF-α and IFN-γ were 2.11 ± 0.14, 13.36 ± 1.97, 143.83 ± 11.92, 55.49 ± 5.94, 9.04 ± 0.01 and 0.55 ± 0.19pg/ml, respectively. In the dry eye group, the initial concentrations of IL-1β, IL-6, IL-8, MCP-1, TNF-α and IFN-γ were 57.78 ± 5.25, 214.73 ± 18.17, 500.30 ± 38.69, 1518.42 ± 143.29, 385.11 ± 21.95, and 96.55 ± 8.19 pg/ml, respectively. In the dry eye group1 day postoperative concentration of IL-1β, IL-6, IL-8, MCP-1, TNF-α and IFN-γ were 95.54 ± 14.82, 500.30 ± 38.69, 1561.69 ± 289.41, 4595.11 ± 359.16, 621.67 ± 43.55, and 266.79 ± 23.54 pg/ml, respectively. In the no dry eye group, 1 day postoperative concentration of IL-1β, IL-6, IL-8, MCP-1, TNF-α and IFN-γ were 51.42 ± 6.05, 119.51 ± 12.19, 443.92 ± 34.51, 2128.74 ± 215.64, 159.73 ± 12.98, and 51.54 ± 6.82 pg/ml, respectively.

**Fig 3:**
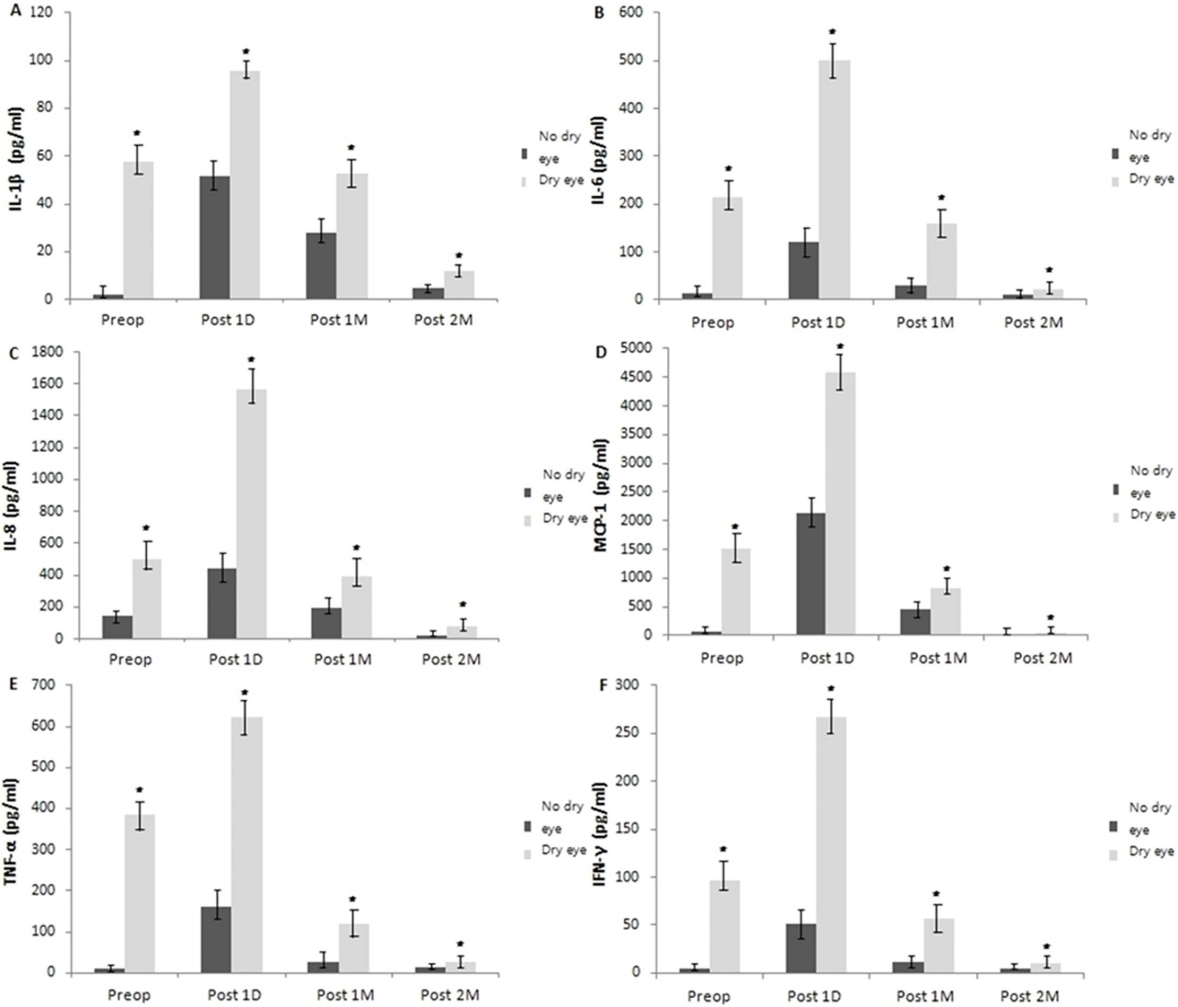
Change in concentration of cytokines and chemokines in lacrimal tears from preoperative value. The decrease in concentrations of IL-1β, IL-6, IL-8, MCP-1, TNF-α and IFN-γ in lacrimal tears was statistically significant in the dry eye group at 1, and 2 months compared to the value of 1 day post-surgery, respectively (*P* <0.05). Each value represents the mean ± standard error of the mean (SEM). Significance was evaluated with random-effects analysis of variance (ANOVA) model; p values of <0.05 were considered statistically significant. ***P < .001, **P < .01, *P < .05, using a linear mixed model with post hoc analysis, or generalized estimating equations model.

In the dry eye group, there was a statistically significant decrease in the concentration of IL-1β (52.49 ± 6.17, and 11.82 ± 5.64, respectively), IL-6 (159.13 ± 19.04, and 22.16 ± 5.91, respectively), IL-8 (394.16 ± 30.81, and 80.62 ± 7.53, respectively),), MCP-1 (815.26 ± 65.49, and 47.15 ± 5.61, respectively), TNF-α (118.17 ± 11.52, and 26.48 ± 3.68, respectively), and IFN-γ (56.12 ± 5.94, and 9.64 ± 1.80, respectively) in tears at 1 and 2 months following cataract surgery compared to the value of 1 day postoperatively (*P* <0.05, respectively) (Fig 3).

Furthermore, in the no dry eye group, there was also a statistically significant decrease in the concentration of IL-1β (27.64 ± 7.21, and 5.02 ± 0.28, respectively), IL-6 (29.48 ± 6.98, and 11.42 ± 1.53, respectively), IL-8 (196.43 ± 12.51, and 21.46 ± 4.63, respectively), MCP-1 (449.75 ± 41.62, and 29.58 ± 5.24, respectively), TNF-α (26.25 ± 2.31, and 13.64 ± 1.98, respectively), and IFN-γ (11.16 ± 1.85, and 5.10 ± 1.02, respectively) in lacrimal tears at 1 and 2 months following cataract surgery compared to the value of 1 day postoperatively (*P* <0.05, respectively). All quantified cytokines and chemokines had a tendency to be increased at 1 day postoperatively and decreased at 1 and 2 months after cataract surgery (Fig 3).

### Evaluation of Meibomian gland

#### 1) Lid margin abnormalities

**Fig 4:**
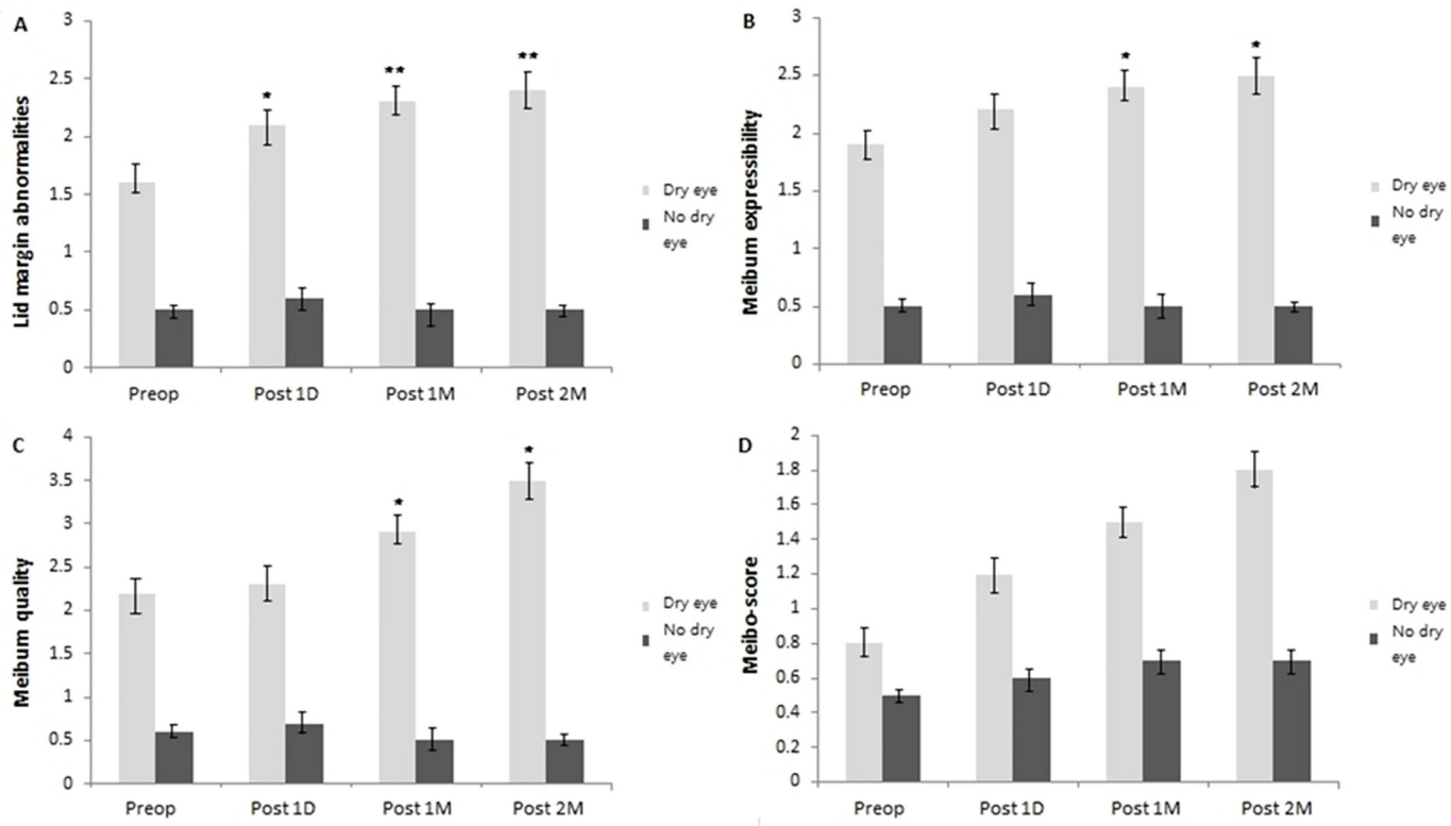
Change in lid parameters of MGD from preoperative value. (A) Change in lid margin abnormality scores from preoperative value. Lid margin abnormality scores were significantly higher in the dry eye group at 1 day, 1 and 2 months postoperatively compared to the no dry eye group (*P* <0.05). (B) Change in meibum expressibility from preoperative value. There was a statistically significant increase in the meibum expressibility at 1 and 2 months postoperatively and the dry eye group showed higher level than the no dry eye group (*P* <0.05). (C) Change in meibum quality from preoperative value. There was a statistically significant increase in the meibum quality at 1 and 2 months postoperatively and the dry eye group showed higher level than the no dry eye group (*P* <0.05). (D) Change in meibo-score from preoperative value. Meibo-score was higher in the dry eye group at 1 day, 1 and 2 months postoperatively compared to the no dry eye group. Each value represents the mean ± standard error of the mean (SEM). Significance was evaluated with random-effects analysis of variance (ANOVA) model; p values of <0.05 were considered statistically significant. ***P < .001, **P < .01, *P < .05, using a linear mixed model with post hoc analysis, or generalized estimating equations model.

There was a statistically significant increase in lid margin abnormalities at 1 month (2.3 ± 0.6, 0.5 ± 0.1, respectively), 2 months (2.4 ± 0.6, and 0.5 ± 0.1, respectively) postoperatively in the dry eye group compared to the no dry eye group (*P* <0.01, respectively) (Fig 4A). It was more significantly worsened in the dry eye group compared to the no dry eye group at 1 day postoperatively. There were aggravations at 1 and 2 months postoperatively but there was not a significant difference between treatment with topical solutions and the month without treatment.

#### 2) Meibum expressibility

The meibum expressibility was significantly worse in the dry group than in the no dry eye group at 1 month (2.4 ± 0.5, 0.5 ± 0.1, respectively) and 2 months postoperatively (2.5 ± 0.6, 0.5 ± 0.1, respectively) (*P* <0.05, respectively) (Fig 4B). It was more significantly worsened in the dry eye group compared to the no dry eye group at 1 day postoperatively. There were aggravations at 1 and 2 months postoperatively but there was not a significant difference between treatment with topical solutions and the month without treatment.

#### 3) Meibum quality

The meibum quality was significantly worse in the dry group than in the no dry eye group at 1 month (2.9 ± 0.3, 0.5 ± 0.1, respectively) and 2 months postoperatively (3.5 ± 0.5, 0.5 ± 0.1, respectively) (*P* <0.05, respectively) (Fig 4C). It was more significantly worsened in the dry eye group compared to the no dry eye group at 1 day postoperatively. There were aggravations at 1 and 2 months postoperatively but there was not a significant difference between treatment with topical solutions and the month without treatment.

#### 4) Meibo-score

The meibo-scores did not change significantly postoperatively than they were preoperatively. The meibo-scores were worse in the dry group than in the no dry eye group at 1 month (1.5 ± 0.7, 0.7 ± 0.2, respectively) and 2 months postoperatively (1.8 ± 0.7, 0.7 ± 0.3, respectively) even though there was no statistically significant difference between the two groups in the mean meibo-scores (*P* >0.05, respectively) (Fig 4D).

### Correlation analyses

Spearman correlation coefficients between tear cytokine concentrations and parameters of the dry eye are presented in Table 2. TBUT was inversely correlated with IL-8, IL-6, IL-1β, IFN-γ, TNF-α and MCP-1 concentrations and Schirmer I scores were inversely correlated with IL-6 concentrations at 2 months postoperatively. The corneal staining scores showed positive correlations with IFN-γ, IL-8, MCP-1 and IL-6 concentrations. The rank (Spearman) correlation coefficients between ocular symptom scores and parameters of MGD are presented in Table 3. Significant correlations were observed between lid margin abnormalities, meibum expressibility, meibum quality and ocular symptom scores at 2 months postoperatively.

**Table 2:** Correlation between cytokines in the lacrimal tear samples and dry eye parameters at 1 month postoperatively.

	TBUT	Schirmer I test	Corneal staining score
**IL-1β**	-0.34	-0.41	0.38
	*0*.04*	*NS*	*0*.03*
**IL-6**	-0.35	-0.31	0.39
	*<0*.01*	*0*.03*	*<0*.01*
**IL-8**	-0.29	-0.25	0.26
	*0*.01*	*NS*	*0*.02*
**MCP-1**	-0.28	-0.25	0.31
	*0*.021*	*NS*	*0*.034*
**IFN-γ**	-0.25	-0.23	0.33
	*0*.04*	*NS*	*0*.03*
**TNF-α**	-0.14	-0.11	0.16
	*0*.032*	*NS*	*0*.027*

Numbers are Spearman correlation coefficients and *p* value (NS: non-specific)

Significant correlations are marked as *

**Table 3:** Correlation between ocular symptom score and MGD parameters at 2 months postoperatively.

	Ocular symptom score	*p* value
**Lid margin abnormality**	0.37	*0*.002*
**Meibum expressibility**	0.21	*0*.031*
**Meibum quality**	0.19	*0*.029*
**Meibo-score**	0.16	*0*.*067*

Numbers are Spearman correlation coefficients and *p* value (NS: non-specific)

Significant correlations are marked as *

## Discussion

After cataract surgery, patients often experience dry eye symptoms such as eye irritations and complain of tear film dysfunction [20]. Oh et al. reported that tear film function was gradually improved but, dry eye symptoms were worse and did not return to normal levels even after 3 months following cataract surgery [15]. Consistent with this report, we also observed a statistically significant increase in ocular symptom scores after cataract surgery.

Liu et al. showed the worsening of dry eye and the scores which were found by TBUT and Schirmer test after phacoemulsification [21]. Consistent with this report, we also observed a significantly decreased value of TBUT and Schirmer I test following cataract surgery.

The corneal sensitivity threshold decreased immediately after cataract surgery but, showed a gradual improvement at 1 and 2 months postoperatively. A possible explanation for this pattern observed in our study may be the revival of the corneal nerves. The clear corneal incisions made during cataract surgery can lower the sensitivity [3]. On postoperative healing, new neurite cells appear and neural growth factors are released to regenerate the corneal axon [22]. This may explain the dry eye, which was observed early after cataract surgery but improved thereafter. Oh et al. reported that the greater incision size is associated with the slower recovery of sensitivity [15]. Microincisional procedures such as phacoemulsification which is commonly performed today are assumed to generate less reduction in corneal sensitivity than conventional cataract surgery [22].

The dry eye is characterized by the alterations in lacrimal tear composition that may be responsible for the irritation symptoms [10]. IFN-γ has been reported to promote goblet cell loss in an experimental murine model of dry eye [23]. In our study, the increase in IFN-γ concentration after cataract surgery was observed which was particularly higher in the dry eye group. This suggests that IFN-γ producing inflammatory cell is mobilized to the ocular surface, especially in the dry eye group. In previous studies increased levels of ribonucleic acid transcripts encoding IL-1, IL-6, TGF-β1, and TNF-α and were detected in the conjunctiva of patients with keratoconjunctivitis sicca [24–26]. Similarly, our study found that parameters of dry eye, such as TBUT and corneal staining scores, revealed significant correlations with the concentration of IL-6 in tears after cataract surgery. Statistically significant correlations were observed between IL-8, MCP-1, IL-6, IL-1β, IFN-γ, TNF-α and TBUT following cataract surgery. Statistically significant correlations were also observed between IL-8, MCP-1, IL-6, IL-1β, IFN-γ, TNF-α and corneal staining scores following cataract surgery, although Schirmer test scores were not correlated with other cytokines except IL-6. Our study also revealed significant differences in the levels of the certain cytokines in the dry eye group compared with the no dry eye group after cataract surgery. Therefore, tear inflammatory cytokines could be targeted to decrease the damage of the ocular surface after cataract surgery.

Many previous studies showed that the inflammation in patients with MGD is associated with the elevated levels of the cytokines in lacrimal tears [27–30]. Especially, increased concentrations of IL-8, IL-6, TNF-α and IFN-γ were reported in MGD [28]. In our study all patients were prescribed topical antibiotics and steroids postoperatively. Therefore, it would have influenced the result which showed decreased concentrations of tear cytokines at 1 and 2 months postoperatively, although lid margin abnormalities and meibum quality were worse following cataract surgery.

The DEWS management and therapy subcommittee reported that any severity levels of the dry eye may have MGD [13]. Modified or deficient meibum makes evaporative type dry eye that are features of MGD [31,32]. Consistent with these reports, in our study the patients who still complain of ocular discomfort, yet the ocular surface inflammation related to the cataract surgery itself not seen by an objective ophthalmologic examination, showed clinical signs of MGD such as vascular engorgements and irregular lid margin. Moreover, the ocular symptom scores did not return to the preoperative level at 2 months postoperatively, which was still high enough to show the ocular discomfort of the patients who had undergone phacoemulsification. Moreover, statistically significant correlations were observed between lid margin abnormalities, meibum expressibilty, meibum quality and ocular symptom scores following cataract surgery. Therefore, ocular discomfort seen after cataract surgery possibly could be caused by MGD. Despite its clinical importance, the influence of the cataract surgery to MGD has not been investigated sufficiently. Our study demonstrated a statistically significant worsening of lid margin abnormality scores, increased meibum quality and expressibility scores after cataract surgery performed by the phacoemulsification. Therefore, it is important to evaluate the meibomian glands preoperatively and postoperatively and pay attention to the intraoperative care carefully.

MGD is diagnosed by slit-lamp examination of the lid margins [33], meibometry [34,35], assessment of the volume and properties of the meibum [36,37], meibography [38–42], assessment of tear interference [43,44], and assessment of the tear evaporation rate [45,46]. However, in our study the power of the meibum quality and expressibility scores were slightly lower than those of ocular symptom scores and lid margin abnormality scores, partially because the pressure of the expression to score meibum is not well defined. When more objective and reproducible methods to score meibum is developed, it may have a higher power than the result of our study to differentiate the patients with MGD from the patients without MGD after cataract surgery.

The cataract surgery might have caused meibomian gland obstruction since functional changes of the meibomian glands were detected in our study accompanying structural changes. Decreased corneal sensations may result in decreased blinking rates which probably make less releasement of the abnormally modified meibum. Lid dysfunction that results from the use of the lid speculum also could cause MGD after cataract surgery. The longer follow up period would elucidate more definite changes of the meibomian glands after cataract surgery.

In our study using the meibography, we showed changes of the meibomian gland after cataract surgery. But, no significant correlation was observed between meibo-score and ocular symptom scores at 2 months postoperatively. Even though the meibo-score did not show a statistically significant difference, meibo-scores were higher in the dry eye group than the no dry eye group. Also, patients with various changes of the meibomian glands were observed, including gland dropout, shortening, distortion and dilation of the proximal portion of the meibomian gland. These findings suggest that the obstruction of the orifices after cataract surgery might be responsible.

A possible explanation for the difference of results between the period with topical therapy (1 day to 1 month postoperatively) compared to the period without topical therapy (month 1 to months 2 postoperatively) may be due to the different eye drops and duration of medications used. Topical steroid eye drop is currently the main treatment in moderate to severe dry eye. Many studies have shown the efficacy of it in treatment of dry eye [47]. The most beneficial effect of steroids is the rapid onset of action which makes them handy in circumstances when immediate anti-inflammatory reaction is needed such as after cataract surgery. Previous study showed that 0.5% loteprednol etabonate had significantly good outcomes after 2 weeks of the treatment [48]. Consistent with this report, our study revealed statistically significant improvements in TBUT, corneal staining score and corneal sensitivity threshold at 1 month postoperatively when topical eye drops were used compared to the period without topical therapy which is the months 2 postoperatively.

In patients with MGD, antibiotics may play a role in the treatment. Topical azithromycin suppresses bacterial lipases, preventing degradation of the normal meibum [49]. Previous study showed that patients with MGD receiving loteprednol etabonate four times daily had marked improvements in meibum quality and TBUT at 1 month [50]. However in our study, antibiotic and steroid used after cataract surgery did not show statistically significant improvements in lid margin abnormalities and MG secretions postoperatively. These results suggest that cataract surgery may influence MG function and exacerbate MG obstruction despite the use of topical antibiotics and steroid. The ocular surface inflammation and occurrence of dry eye followed by cataract surgery, lid dysfunction resulting from the use of a lid speculum, and decrease in blink rate derived from a decrease in corneal sensation may influence MG function after cataract surgery. Further study would be beneficial which could discriminate the effect of the surgery from the effect of antibiotics and steroids.

This study was conducted on a relatively small number of patients that was powered to detect differences of the meibomian glands and tear film instability before and after cataract surgery. Despite this design, significant differences were observed in the levels of cytokines and MGD parameters in the dry eye group compared with the no dry eye group.

Irritating symptoms are common complaints of the patients after cataract surgery seeking medical consultation. Even though the changes of various ocular surface parameters and tear film instability that was worse immediately after cataract surgery returned to the preoperative level at postoperative 2 months, the morphologic and functional changes of the meibomian glands emerged following cataract surgery which were correlated with the increased ocular symptom scores. Therefore, these could elucidate the development of dry eye related to cataract surgery. It is important to assess meibomian gland dysfunction after cataract surgery to avoid damage to the ocular surface and to ensure high quality of vision.
